# Long-Term Stability Improvements of the Miniature Atomic Clock Through Enhanced Thermal Environmental Control

**DOI:** 10.3390/s25185817

**Published:** 2025-09-18

**Authors:** Emily Gokie, Jon Omaraie, Thejesh N. Bandi

**Affiliations:** 1 Quantime Lab, Department of Physics and Astronomy, The University of Alabama, Tuscaloosa, AL 35487, USA; ejgokie1@crimson.ua.edu (E.G.); jomaraie@crimson.ua.edu (J.O.); 2 Time and Frequency Division, National Institute of Standards and Technology, Boulder, CO 80305, USA

**Keywords:** miniature atomic clock (MAC), chip-scale atomic clock (CSAC), alternate-PNT, space navigation

## Abstract

Advancement of compact atomic clocks has centered on reducing footprint and power consumption. Such developments come at the cost of the clock’s stability performance. Various commercial and military applications demand reduced size, weight, and power (SWaP) requirements but desire an enhanced stability performance beyond what is achieved with the lower-profile standards, such as Microchip’s chip-scale atomic clock (CSAC) or miniature atomic clock (MAC). Furthermore, a high-performing space-rated clock will enhance small satellite missions by providing capability for alternate PNT, one-way radiometric ranging, and eventual lunar PNT purposes. The MAC is a strong candidate as it has modest SWaP parameters. Enhanced performance improvement to the MAC, particularly in the medium to long-term stability over a day and beyond will strengthen its candidacy as an on-board clock in small satellite missions and other ground-based applications. In this work, using external thermal control methods, we demonstrate an improvement of the MAC performance by at least a factor of five, showing a superior stability of σ_y_ = 4.2 × 10^−13^ compared to the best-performing miniaturized standard on the market for averaging intervals of *τ* > 10^4^ s up to 4 days.

## 1. Introduction

Atomic clock technology has revolutionized ground and space applications that require precision positioning, navigation, and timing (PNT) [1,2,3]. The development of coherent population trapping (CPT) coupled with advancements in MEMS devices has enabled the production of compact atomic clocks [4,5]. The advent of miniature atomic clocks (MACs) and chip-scale atomic clocks (CSACs) that operate with lower size, weight, and power (SWaP) requirements while maintaining superior long-term performances over their crystal oscillator counterparts has strengthened the PNT field [6]. MACs and CSACs are now ideal candidates for situations in which the stability of an atomic frequency standard was previously inaccessible. Current application of the compact atomic clocks includes ground-based operation as holdover devices for telecommunications base stations [7] and implementation in GNSS receivers for improved positioning performance and security against spoofing and jamming attacks [8,9,10]. Now the military, space agencies, and commercial developers look to the implementation of the devices on small satellites for network synchronization, alternate-PNT, and navigation purposes.

The space environment poses several challenges to the operation of the compact clocks; extreme temperature fluctuation is at the forefront of the challenges to clock performance [11]. The impact of thermal cycling on clock stability is evident at the timescales corresponding to a fraction of the satellite’s orbital period and beyond, which can range from 90 min for a satellite in LEO to 12 h for a satellite in MEO. The overall temperature variation experienced by the electronics aboard small satellites will depend on spacecraft design and orbit type, but the relatively small mass of the spacecraft makes it susceptible to changes in thermal flux [12]. Thermal analyses of small satellites found that temperature swings on the order of tens of degrees are experienced throughout their orbit [13]. These large temperature fluctuations result in significant degradation in long-term stability (averaging intervals *τ* > 10^3^ s) of the clocks due to their thermal sensitivity. The instability arising from this thermal sensitivity critically impacts the ability of the spacecraft to operate independently of GNSS or ground-based corrections.

Precise onboard timekeeping for autonomous navigation was marked as a critical technology by the International Space Exploration Coordination Group in their Global Exploration Roadmap [14]. The navigational precision for spacecraft that rely on measurements made from an onboard clock (one-way ranging) is determined by the induced positioning error from clock instability. The listed requirement for absolute position knowledge is <0.4 m (GER-027) with an emphasis on longer intervals between synchronization with ground stations. Multiple studies have characterized the performance of the CSAC as a candidate for one-way radiometric ranging through simulations and technology demonstration missions. Simulations of CSAC-equipped CubeSats in LEO and lunar orbit demonstrate positioning estimation errors in the orbit determination solution on the order of hundreds of meters—these errors are greatly exacerbated by the degraded clock performance from the temperature variation seen in the orbital period [15,16]. A NASA demonstration mission of a CSAC paired with a software-defined radio in lunar orbit showed that the orbit determination solution had positioning estimation errors up to 10 km, an order of magnitude degraded from traditional two-way tracking techniques [17].

The positioning performance utilizing the chip-scale atomic clock demonstrated in these studies is suitable for missions with moderate navigation constraints. Given that the onboard clock was a major contributor to the positioning error, a higher-grade standard will expand the capabilities of one-way radiometric ranging to low-SWaP missions with more rigorous navigation demands. A low SWaP standard with excellent long-term stability will also be necessary to meet synchronization requirements in communication and PNT networks operating independently of GNSS. The higher-profile miniature atomic clocks generally offer an order of magnitude performance improvement to the chip-scale standards. However, this level of performance is still undesirable for many navigation and timing applications and is further degraded by sensitivity to temperature fluctuations. Accordingly, further enhancements to long-term stability can be achieved through the limitation of environmental temperature variation.

To the best of our knowledge, no attempt has been made to incorporate environmental thermal control measures to enhance stability performance in a miniature atomic clock. The impact of temperature cycling on the chip-scale atomic clock has been simulated; the studies of CSAC-equipped CubeSats simulated the deterioration of stability with thermal cycling for estimating the clock signature [15,16]. These results address a method to model thermal effects in the CSAC, but they also show that such thermal cycling cannot be ignored in clock performance. In terms of hardware testing, Microchip released a whitepaper on the performance of the CSAC during a rapid temperature change [11]. This study displays the frequency shift induced by a temperature shift for several CSACs, but it is not focused on thermal confinement or control. Furthermore, these efforts have been focused on the smaller profile, lower-grade chip-scale atomic standards and fail to address impacts on the higher-grade miniature atomic clock. This is the first work to demonstrate the feasibility of applied thermal control on the stability performance of any low SWaP clock, such as a miniature atomic clock.

This work quantifies the stability improvement of Microchip’s MAC SA.35m with enhanced thermal control. The studies performed in this paper utilize simple PID control and thermal isolation techniques. The added size, weight, and power associated with the precise temperature control are evaluated along with performance improvement. The reviews in Section 1 and Section 2 demonstrate the applications that will benefit from the superior performance.

Section 2 of this paper evaluates and compares the predicted positioning error of the commercially available low-SWaP standards and details this implication in small satellite PNT purposes. Section 3 provides an overview of the measurement methods and the implementation of the thermal control systems for this work. Section 4 analyzes the stability performance of the MAC SA.35m with the applied thermal control. Section 5 then evaluates the positioning performance with the stability improvement from thermal control, quantifies the added SWaP of a precise thermal control setup, and discusses the impact on timing and positioning applications. Finally, Section 6 outlines future work.

## 2. Positioning Error of the Low-SWaP Clocks

The estimated positioning error from a clock at any time *t* can be calculated as the product

(1) $$\sigma_{\rho }(t)=cx(t),$$

where *c* is the speed of light and *x*(*t*) is the predicted time error of the clock. The predicted time error accumulated after an initial synchronization is given as

(2) $$x\left(t\right){=x}_{0}+y_{0}t+\frac{1}{2}Dt^{2}+\int_{0}^{t}y_{T}(t)dt+\varepsilon (t),$$

where *x*_0_ is the initial time offset, *y*_0_ is the initial frequency offset, *D* is the linear frequency drift, *y_T_*(*t*) is the environmentally induced frequency offset and *ε*(*t*) is the time error induced by the purely stochastic clock processes [11]. The induced frequency offset from environmental perturbations is taken to be

(3) $$y_{T}(t)=y_{T,o}(t)+\delta y_{T}(t),$$

where $y_{T,o}$ is a known thermally induced frequency offset and $\delta y_{T}(t)$ is the non-predictable frequency offset from stochastic variations in temperature. Now the predicted time error from the random frequency instabilities in Equation (2) are grouped as

(4) $$\sigma_{x}\left(\tau \right)={\left[\langle \varepsilon^{2}\left(\tau \right)\rangle +Var\left[\int_{0}^{\tau }\delta y_{T}\left(t\right)dt\right]\right]}^{1/2}.$$

This term combines the offset induced by stochastic temperature variations as well as the offset from purely stochastic processes independent of the environment. The random predicted time error can be characterized with the Allan Deviation (ADEV), *σ_y_*(*τ*) [18]. This term is dependent on the power-law noise type that dominates in a given averaging interval, *τ*. For averaging periods sufficiently shorter than the length of the data set and the removal of known frequency offset and drift from the data, the estimate for the predicted time error is

(5) $$\sigma_{x}\left(\tau \right)=k\tau \sigma_{y}(\tau ),$$

where *k* is the coefficient dependent on the governing noise type for the averaging period. The dominant noise type is easily observed from the slope of the clock’s Allan Deviation [19]. The value of this coefficient for the given noise type and corresponding ADEV slope is displayed in Table 1.

Since the coefficient is unity for white frequency noise and the time error from the drift term in Equation (1) is negligible at small timescales, the time error over a given averaging interval is approximately taken as *τ.σ_y_*(*τ*) when estimating clock performance. However, the focus of this work is on clock behavior over longer timescales (*τ* > 10^3^ s), where drift and flicker frequency can eventually become dominant. Thus, the resulting positioning error over a specified averaging interval for an oscillator with zero phase and frequency offset can be calculated as

(6) $$\sigma_{\rho }(\tau )=c\left[\frac{1}{2}D\tau^{2}+\sigma_{x}(\tau )\right]$$

where the values of the coefficient *k* are taken from Table 1. From Equation (6), the dependence of positioning performance on clock stability and averaging interval is clearly seen. In some cases, where the drift of the oscillator can be observed [20], the first term in Equation (6) can be corrected for.

Now, an evaluation and comparison of the positioning performance of low-SWaP atomic clocks is performed here utilizing Equation (6). The operating parameters, thermal sensitivity, and calculated positioning error of the commercially available chip-scale and miniature atomic clocks are compared in Table 2.

These clocks are characterized as chip-scale atomic clocks (lower profile and lower grade) or as miniature atomic clocks (higher profile and higher grade). It is relevant to note that only two of the commercially available low-SWaP atomic clocks are currently space-rated. A space-rated CSAC was first developed by Microchip [21]. Safran has also released a rubidium standard, the mRO-50, for aerospace application [22].

**Table 2:** Comparison of SWaP, thermal sensitivity, and performance of several of the chip-scale and miniature atomic clocks.

Clock Type	Size [cm^3^]	Weight [g]	Power [W]	Temperature Range [°C]	Thermal Sensitivity	Averaging Interval *τ* [s]	ADEV	Positioning Error [m]
chip-scale atomic clocks								
Microchip CSAC SA.45s [23]	17	35	0.12			10^1^	1.00 × 10^−10^	0.30
				−10 to 70	<5 × 10^−10^	10^2^	3.00 × 10^−11^	0.90
						10^3^	1.00 × 10^−11^	3.00
Teledyne CSAC [24]	23	43	0.18	−10 to 60	<5 × 10^−10^	10^1^	1.00 × 10^−10^	0.30
						10^2^	3.00 × 10^−11^	0.90
						10^3^	1.00 × 10^−11^	3.00
Spaceon XHTF1045 CSAC [25]	17	35	0.25	−40 to 70	<1 × 10^−9^	10^1^	1.00 × 10^−10^	0.30
						10^2^	3.00 × 10^−11^	0.90
						10^3^	N/A *	-
AccuBeat NAC2 [26]	24	38	0.35	−40 to 80	<5 × 10^−10^	10^1^	8.00 × 10^−11^	0.24
						10^2^	2.00 × 10^−11^	0.60
						10^3^	1.00 × 10^−11^	3.00
miniature atomic clocks								
Microchip MAC SA.35m [7]	50	86	5			10^1^	1.60 × 10^−11^	0.05
				−10 to 75	<1 × 10^−10^	10^2^	8.00 × 10^−12^	0.24
						10^3^	N/A *	-
Microchip MAC SA.57m [27]	47	100	6.3	−40 to 75	<2 × 10^−11^	10^1^	5.00 × 10^−12^	0.02
						10^2^	1.50 × 10^−12^	0.05
						10^3^	5.00 × 10^−13^	0.19
Safran mRO-50 [22]	50	75	0.45			10^1^	3.00 × 10^−11^	0.09
				−10 to 60	<4 × 10^−10^	10^2^	1.00 × 10^−11^	0.30
						10^3^	N/A *	-

* Values not specified in the manufacturer’s datasheet.

The positioning error induced by the instability of the miniature atomic clocks is over an order of magnitude less than that of the chip-scale atomic clocks at the longer timescales. However, no space-rated standard with the stability performance and profile of the higher-grade MAC has been released. There exist many functions that would benefit from a stable clock with a small footprint, but are not so conservative as to require the tight operating conditions of the chip-scale atomic clocks. The long-term stability of the low-SWaP standards as it stands is insufficient to achieve the autonomous navigation capability outlined in the Global Exploration Roadmap [14]. Thus, in the implementation of an enhanced thermal control scheme that will improve the stability of the clocks as well as add some weight and power to the setup, the choice of the already higher-grade, higher-profile miniature atomic clocks is ideal. The remainder of this section examines the expected performance of low-SWaP clocks in notable Earth orbit and cis-lunar PNT applications, evaluating the need for further long-term improvements, precise thermal control, and SWaP constraints.

Distributed small satellite systems are an active area of research for complementary PNT purposes. Recently, the U.S. Space Force created the Resilient GPS (R-GPS) program to augment the current GPS medium Earth orbit (MEO) constellation with several smaller satellites [28]. To meet the operational requirements of these satellites, a stable low-SWaP clock will be necessary. The satellites in this orbit maintain a constant ground track, allowing resynchronization with a ground station every 12 h (*τ* ≈ 4 × 10^4^ s). The onboard clock therefore, must perform optimally out to these timescales. While the satellites in these orbits experience fewer occultation periods with the Earth, the thermal variation is also more extreme than in LEO [29]. The award per satellite was approximately one-fifth of the cost of a Block III satellite. Thus, the mass and operating parameters of the augmented satellites will be reduced by a similar fraction compared to the traditional GPS satellites, which range from 1700 to 2200 kg. Assuming a weight reduction proportional to the cost, these satellites will still carry a mass upwards of 200 kg, making the employment of enhanced thermal control measures feasible for the clocks.

Independent of GPS, the development of synchronized small satellite networks using precise clocks and optical crosslinks (and time transfer) for alternate-PNT is of interest for military and commercial applications [30]. Optical crosslinks are an emerging technique in spacecraft communication and navigation. Spacecraft-to-spacecraft optical time transfer via a laser link will be demonstrated with the upcoming CubeSat Laser Infrared Crosslink (CLICK) mission [31]. While optical time transfer will provide a method for LEO satellites to make corrections in lieu of GNSS corrections, the longer-term stability of such satellites becomes critical. Laser time transfer methods require precise pointing, acquisition, and tracking of targets. Thus, to reduce the burden on such a system and allow for longer periods between synchronization events, the onboard clock must maintain its stability over these intervals. MACs and CSACs have already been modeled as the reference for lasing measurements in small satellite PNT networks [32], but a clock with further enhanced stability will improve performance. Satellites in these networks will experience large thermal cycling given the regular Earth occultation in LEO [29], making temperature stabilization particularly relevant for thermally sensitive electronics. CubeSats pose a unique challenge in the implementation of additional thermal control measures due to their stringent size, weight, and power requirements. However, the achievement of precise thermal control has been demonstrated for sensitive equipment [33]. Future LEO satellite constellations consisting of CubeSats therefore must consider the tradeoff of synchronization and performance and the SWaP of the clock and thermal system.

Outside of Earth orbit, low-SWaP clocks are potential candidates in cis-lunar and deep space navigation applications. As a method of providing communication and positioning services on the moon, NASA’s Goddard Space Flight Center has proposed a network of lunar-orbiting satellites [34]. Compact atomic clocks, such as the CSAC and MAC, are leading onboard clock suggestions due to their minimal operating parameters. A requirement for the performance of this PNT service is a ±10 m for surface operations (LCRNS.3.0570) [35]. The stability of the onboard clock directly affects this positioning error. A case study on the design of such a lunar constellation with timing corrections supplemented by the Earth-based GPS demonstrated that the MAC resulted in approximately half the lunar User Equivalent Ranging Error (UERE) as the CSAC for all orbit types [36]. The timing errors in these computations primarily depend on the length of the period in which the satellite has no visibility with GPS, up to an interval of *τ* = 3.9 × 10^3^ s for the studied orbits. Given the large surface temperature variations on the moon [29], the thermal load on a satellite can vary significantly throughout an orbit [37]. Thus, to ensure that errors from clock instability remains low during the long outage intervals, the application of precise thermal control should be considered. Given the goal of providing lunar navigation services in a fashion similar to GNSS, an enhancement to the clock payload is essential to ensure the precision of the PNT service. The mass of these satellites (<180 kg) is substantial enough that tradeoff in SWaP is less significant than on the more stringent CubeSat missions. Several other proposed lunar PNT schemes also note the critical impact of clock performance on positioning performance [38,39], further demonstrating the relevance of optimal clock stability in the thermally varied lunar environment.

The above positioning and performance requirements demonstrate the importance of the long-term stability of the onboard clock in LEO, MEO, and lunar small satellite missions. The current limitations and thermal sensitivity of the low-SWaP clocks must be addressed to meet these performance goals and enhance mission autonomy. Enhanced thermal management of the low-SWaP clocks, particularly in the higher-grade miniature atomic clocks, is a method to address these limitations in their long-term stability.

## 3. Methodology

Perturbation due to thermal variation is a major limiting factor for a clock’s long-term stability. The stability of most present day low-SWaP commercial atomic clocks is tied to the measurement of a microwave resonance frequency between two hyperfine ground states of an alkali atom (rubidium for the MAC and cesium for the CSAC). Temperature changes in the clock’s physics package affect stability in at least two distinct ways: the temperature dependence of buffer gas collision frequency shifts and the influence of thermal gradients on vapor number densities [5]. Additionally, the cavity laser’s frequency is dependent on laser temperature and injection current. Changes to the laser temperature result in changes to the laser injection current in order to keep the laser on resonance. As this injection current changes, it causes laser intensity variations that result in changes in the AC Stark Shift. These processes degrade the clock performance. Thus, the employment of more stringent thermal control to the device will result in an improved frequency stability.

**Figure 1:**
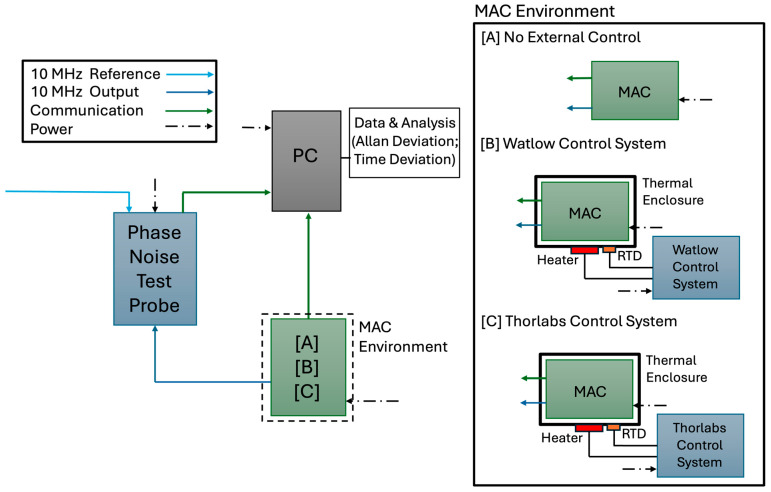
The setup of the MAC performance testing for the conditions of [A] no external control, [B] control with the Watlow system, and [C] control with the Thorlabs system.

Microchip’s legacy MAC SA.35m was used for our studies. To determine the improvement in the performance of the MAC from varying levels of thermal stability, two types of thermal control methods were implemented in addition to the initial characterization of the device. Figure 1 shows the setup of the MAC performance testing in a thermal control box that was designed and built. Three different thermal conditions were tested: without external control, with the Watlow control system, and with the Thorlabs thermal control system.

The preliminary characterization of the MAC was conducted in its ambient environment. This was necessary as the manufacturer’s data is only available for three averaging times (all *τ* < 10^2^ s), and individual instrument performance shows some variation from the specified values. The first thermal confinement test was designed to achieve an intermediate level of thermal control. A system with a Watlow F4DH Controller [40] was constructed and deployed to gauge clock performance. The datasheet temperature stability of this device for the testing conditions is ±0.75 °C. In testing, the controller achieved a better performance with a measured temperature stability of ±0.034 °C (1-sigma). These tests characterized MAC performance with moderate improvements to thermal sensitivity. For an improved thermal stability, a control system utilizing a Thorlabs TC300B Temperature Controller [41] was implemented. The datasheet temperature stability of the Thorlabs controller is ±0.1 °C, and its measured temperature stability was ±0.025 °C (1-sigma). These tests characterized MAC performance with further improvements to its thermal sensitivity. The remainder of this section is dedicated to the setup for each system.

### 3.1. Ambient Characterization of the MAC

Prior to the application of external thermal control, several stability performance tests were conducted to characterize the individual behavior of the clock. While the performance values for the device are provided in the manufacturer’s datasheet for shorter time periods, individual characterization is necessary to obtain instrument-specific values as studied in this work. This is necessary to evaluate the long-term performance of the clock, which is of interest to this study. The preliminary performance runs were conducted with the MAC situated on its evaluation board and exposed to the ambient environment at 23 ± 2 °C. This setup is shown as configuration [A] in Figure 1. Several tests were conducted and compared with the Allan Deviation values from the manufacturer’s datasheet. The MAC performed better than the stated datasheet parameters, but its performance was consistent across each run. Thus, the results reflect only one of these preliminary characterization runs.

The overlapping Allan Deviation was used to evaluate clock performance [19]. This metric is the standard measure of clock stability and characterizes the performance of the device from its noise effects, independent of systematic errors, such as a constant temperature offset. A Symmetricom 3120A Phase Noise Test Probe (Symmetricom, San Jose, CA, USA) was used to measure phase variations in the MAC SA.35m with reference to an SRS FS725 Rubidium Frequency Standard (Stanford Research Systems, Sunnyvale, CA, USA). We initially used this standard as a reference for the phase noise measurements due to its ultra-low phase noise (−130 dBc/Hz at 10 Hz offset), one second Allan Variance (<2 × 10^−11^), and monthly drift (<5 × 10^−11^). Furthermore, to overcome the long-term limitations due to FS725, we used Cesium 5071A as a reference clock. This reference clock has excellent stabilities of <1.2 × 10^−11^ at 1 s down to <8.5 × 10^−14^ at 10^5^ s, with a flicker floor <5 × 10^−14^.

The largest temperature fluctuations experienced by the clock occur due to diurnal cycles. Thus, the largest frequency dispersion in the clock due to this cyclical environmental temperature variation will be most extreme around 12 h intervals (*τ* ≈ 4 × 10^4^ s). A performance run of 7 days in length yields an Allan Deviation with sufficiently low uncertainty for this averaging time. Accordingly, all performance runs contained in this paper are at least 7 days in length.

### 3.2. Enhanced Thermal Management

**Figure 2:**
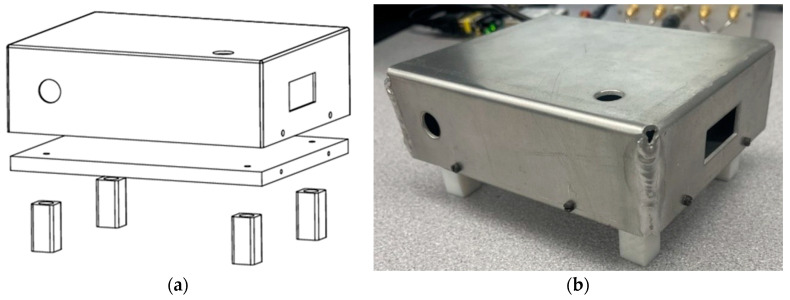
(**a**) The CAD model of the thermal enclosure, and (**b**) the fabricated unit.

An aluminum thermal enclosure was designed and fabricated to fit the MAC and its evaluation board. The enclosure shielded the MAC from any direct air currents and stood on insulative PTFE legs to isolate the clock from the ground platform. The enclosure is shown in Figure 2. A CHOTHERM thermally conductive pad was placed at the interface of the MAC’s evaluation board and the enclosure baseplate to prevent metal-to-metal thermal contact resistance. Two 7 W thin-film heaters were mounted on the bottom of the baseplate to be operated through one of the external control systems. This enclosure was used for both tests with the Watlow controller and the Thorlabs controller.

#### 3.2.1. Watlow Thermal Control System

Characterization of the clock with moderate thermal control will demonstrate the performance improvement of the clock with modest developments to its thermal environment. The relatively small footprint, low cost, and limited temperature stability of the Watlow F4DH controller (Watlow, Winona, MN, USA) made it an ideal device for this demonstration.

The Watlow controller functioned as a PID device. It received a temperature input from a PT100 RTD (Thorlabs, Newton, NJ, USA) mounted in the vicinity of the heaters on the enclosure baseplate. This setup is shown as configuration [B] in Figure 1. Based upon the programmed PID bands and the setpoint of the system, the Watlow unit switched an external DC SSR in the circuit with the heaters and their power supply. Since no cooling technique was implemented in the system, a setpoint temperature was chosen above the nominal baseplate operating temperature so that it could be maintained in the case of an increase in ambient temperature. The temperature-induced frequency shift in the device arising from this temperature offset will not be reflected in the performance runs as the Allan Deviation removed any bias in the run.

#### 3.2.2. Thorlabs Thermal Control System

To demonstrate the performance of the MAC with more rigid thermal confinement, the Thorlabs TC300B Temperature Controller (Thorlabs, Newton, NJ, USA) was chosen for the tests. This characterization will serve as a baseline for the improvement in stability from precise control or enhanced isolation in the clock’s thermal environment.

Unlike the Watlow controller, the Thorlabs controller provided the capability to directly power and switch the heaters, so no external SSR was necessary. This setup is shown as configuration [C] in Figure 1. We note that a simple thermal controller using an Arduino controller can be designed to bring the temperature control down to 1 mK levels. In the same method outlined in Section 3.1, the stability performance of the MAC was measured over runs at least 7 days in length for all tests.

## 4. Results

Three stability performance runs are compared, corresponding to the thermal confinement scenarios outlined in Section 3: ambient characterization, characterization with moderate thermal control with the Watlow controller, and characterization with a tight thermal control with the Thorlabs controller. The tests range from 7 to 21 days in length. The longer duration tests reduce the uncertainty on a given averaging interval [19], but the averaging intervals of primary interest (*τ* > 10^3^ s) have sufficient statistical confidence for comparison across all runs. The temperature fluctuations in the MAC are first discussed for each run before the stability data is presented and performance improvement quantified.

**Figure 3:**
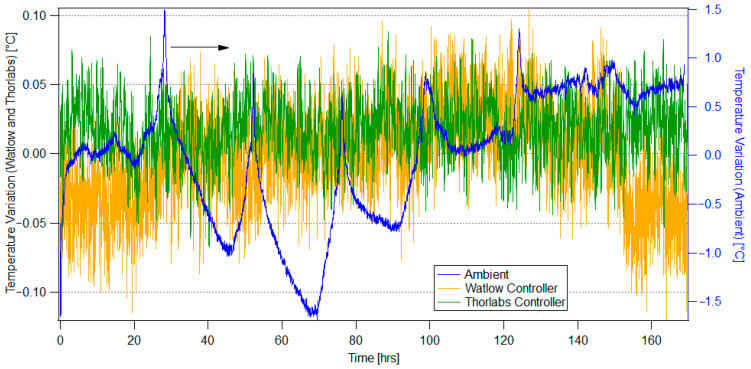
MAC internal case temperature over 7 days for the ambient characterization (blue), characterization with moderate confinement with the Watlow unit (yellow), and characterization with rigorous confinement with the Thorlabs unit (green).

The internal temperature variation from each MAC characterization test is displayed in Figure 3. In the ambient test without applied thermal control, the MAC experienced an overall temperature range of 3.19 °C (1-sigma stability of ±0.66 °C).

As predicted for this case, the temperature variation is cyclical with 24 h periods. The maxima occur around 3:00 in the afternoon. Two reasons can be attributed to this. The first is that the laboratory in which the tests are conducted is in the Southwest corner of the building, with windows on an outer wall that experience direct sunlight mid-afternoon. The second is that this time corresponds to when the most lab members are at their stations, elevating the overall room temperature.

Both thermal confinement tests demonstrate over an order of magnitude reduction in the overall temperature variation in the MAC. Figure 3 displays the difference in temperature stability achieved by the two controllers. The test of moderate thermal control with the Watlow controller displayed a temperature stability of ±0.034 °C. The test of more rigorous thermal control with the Thorlabs controller demonstrates a temperature range of ±0.025 °C.

**Figure 4:**
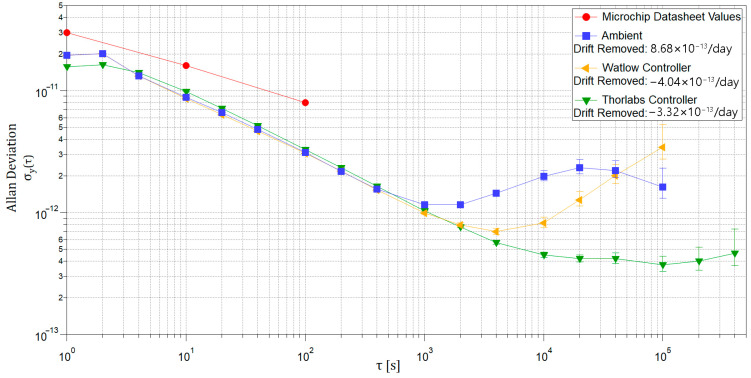
The stability performance of the MAC for ambient characterization (blue), characterization with moderate confinement with the Watlow unit (yellow), characterization with rigorous confinement with the Thorlabs unit (green), and datasheet values (red).

The stability performance for the three thermal control modes is shown in Figure 4 along with the manufacturer’s listed datasheet values. The MAC performed better than the datasheet values across all levels of thermal confinement. However, the manufacturer’s values are only available out to an averaging interval of *τ* = 10^2^ s, limiting a comparison at longer intervals.

All runs displayed a similar performance at averaging intervals of *τ* < 10^3^ s. This region of the Allan Deviation curve reflects white frequency noise (behavior following *τ*^−1/2^) and thus would not be expected to exhibit change as a result of reduced temperature variation. Beyond an averaging period of *τ* = 10^3^ s, the ambient characterization of the MAC (blue) exhibits behavior that reflects stability disturbances from periodic environmental perturbations; the notable bump in its stability curve that peaks between *τ* = 2 × 10^4^ s (~6 h) and *τ* = 4 × 10^4^ s (~12 h) can be attributed to the cyclical thermal variations in its environment. The stability performance of the MAC in both cases of enhanced thermal control (yellow and green) shows significant improvement over the ambient characterization beyond *τ* = 10^3^ s. This indicates that the thermal environment of the MAC has a substantial impact on its performance and is effectively remedied by the applied thermal control.

Moderate thermal control of the MAC using the Watlow controller (yellow) improved its stability by a factor of three over its ambient performance, down to a minimum of σ_y_ = 7.0 × 10^−13^ at an averaging period of *τ* = 4 × 10^3^ s. It is interesting to note that beyond *τ* = 10^4^ s, the Watlow control case demonstrates random-walk frequency noise that does not appear in either the ambient case or the Thorlabs control case. Since the random-walk noise is absent in this data, its source cannot be attributed to the laboratory environment or the experimental setup, suggesting instead that the source is in the Watlow heater itself.

Application of more rigorous thermal control using the Thorlabs controller (green) improved the stability of the MAC by a factor of five over the ambient case, down to σ_y_ = 4.2 × 10^−13^ for an averaging period of *τ* = 4 × 10^4^ s up to four days. As discussed in Section 1, clock performance at these timescales is relevant for autonomous spacecraft operation in LEO, MEO, and deep space. The impact of these results on positioning precision applications is discussed in Section 5.

## 5. Discussion

As per our knowledge, this is the first demonstration of the long-term performance of the MAC (*τ* > 10^3^ s) improved with tighter thermal control, as shown in Figure 4. The tightest level of temperature stability (with the Thorlabs controller) demonstrated an improvement in thermal control by a factor of 17 over the ambient case and an improvement in the clock stability by a factor of five. This behavior displays the linear relationship between temperature stability and clock stability. The remainder of this paper exclusively discusses the ambient case and the tightest thermal control case as they demonstrate the performance potential of a MAC equipped with enhanced temperature stability. The results of these characterizations and the impact of thermal control on performance are assessed in two ways: the effect on deterministic drift and the effect on the stochastic frequency fluctuations (characterized with the ADEV).

The effect of frequency drift on timing and positioning performance is shown in the first term of Equation (6) in Section 2. The impact of enhanced thermal control on drift and related positioning performance is shown in Table 3. It is important to note that the linear drift component is not the only limitation via thermal impact; the cyclic thermal variations cause a significant limitation that is not nullified by the linear drift removal. Therefore, a well-controlled thermal regulation reducing the cyclic variations is a key factor to be considered, as demonstrated in the performance curve shown in Figure 4. In the case of applied thermal control, drift was reduced by more than a factor of two compared with the ambient behavior.

The effect of the non-predictable frequency fluctuations (from both thermally induced fluctuations and clock processes) on performance is characterized by the second term in Equation (6). The results of these characterizations are compared in Table 4 with the datasheet values of the legacy MAC SA.35m (the clock utilized in this work) and the datasheet values of the MAC SA.57m, the top-performing miniature atomic clock. The positioning error induced by the clock instability is calculated from the second term in Equation (6), so that the deterministic frequency drift is omitted. As detailed in Section 2, the estimate in the time error from the stochastic terms in Equation (2) is weakly dependent on the dominant noise type for a given averaging interval (determined from the slope of the Allan Deviation curve). The processing software computed these slopes, and the values were correlated to coefficient *k* in Table 1 for optimum estimation. For the datasheet values, white frequency noise was assumed for *τ* < 10^3^ s and random walk frequency noise was assumed for *τ* > 10^4^ s. The value of *k* for both of these noise types is unity from Table 1.

For timescales *τ* > 10^4^ s, the thermally controlled MAC outperformed Microchip’s highest-performing MAC by a factor of three and outperformed its ambient characterization by a factor of five, achieving an Allan Deviation down to σ_y_ = 3.7 × 10^−13^ at *τ* = 10^5^ s. The excellent performance of the thermally enhanced miniature atomic clock at these timescales displays its potential for implementation on small satellites in view of networking and PNT applications. However, an assessment of the added size, weight, and power requires a precise thermal control scheme must also be addressed with the assessment of their performance improvement. This work implemented simple external PID control with enhanced thermal isolation of the clock to achieve a ±0.025 °C (1-sigma) level of temperature stability. While the system utilized in this setup is not intended to be directly integrated with satellite clock payloads, any thermal control system that achieves a similar level of temperature stability would expect the corresponding performance improvement. The discussion now turns to quantifying the SWaP and addressing the feasibility of such a setup on small spacecraft.

The thermal control system in this work implemented enhanced thermal isolation of the MAC and PID control of resistive heaters adhered to the baseplate of this enclosure. From Table 2, the MAC is 17 cm^3^, 35 g, and consumes 5 W. However, these values do not contain the SWaP of its evaluation board, which was necessary for characterization in these studies. The evaluation board occupied a footprint of (12.7 cm × 9.7 cm) with a power consumption of 15 VDC. The footprint of the Aluminum enclosure, which encompassed both the MAC and its evaluation board, was (14.6 cm × 11.6 cm × 4.6 cm) for a volume of 780 cm^3^. The mass of the enclosure was 0.5 kg. To sustain a temperature elevated approximately 15 °C above ambient in the laboratory conditions, the power consumption of the heaters was nominally 1 W.

Small satellite missions with sensitive instruments have demonstrated thermal control at the level addressed in this work. The ASTERIA LEO CubeSat demonstrated ±0.01 °C thermal stability utilizing thermal isolation methods and resistive heaters operating with PID control [33]. The testing of another system utilizing a graded approach with MLI and PID-controlled compensation heaters mounted to an electronics box achieved a ±0.025 °C level of stability [42]. Both of these examples use similar thermal methods to this work.

Several SWaP parameters are considered in the implementation of thermal control on a spacecraft: the heater power and radiator size associated with maintaining temperature to a desired range, the added weight associated with thermal isolation, and the SWaP associated with the addition of electronics for control. In order to keep electronics in the necessary temperature range throughout an orbit, most spacecraft incorporate mechanical thermostats to control heaters. Thus, the heater power expenditure and radiator size in the general case of these coarsely controlled thermostats will be comparable to a case with tighter control. Of course, these expenses will depend on orbit type, beta angle, and other mission-specific parameters. Sensitive electronics packages should incorporate further thermal isolation techniques, such as multilayer insulation (MLI) blankets or heat switches. The mass of 15-layer MLI blankets is generally around 600 g/m^2^ [43]. As an example, the weight associated with insulating the thermal enclosure (0.5 kg) in this setup would be ~25 g. Microcontrollers (typical operating parameters under 2 W, 50 g) are capable of executing simple PID control schemes, limiting the SWaP added with onboard processors and PCBs. While these components may be difficult to implement on the small CubeSat missions, which range from 1 to 24 kg, they account for a much smaller portion of the budget in other spacecraft, such as the proposed R-GPS satellites or future lunar PNT satellites.

## 6. Conclusions and Future Work

**Figure 5:**
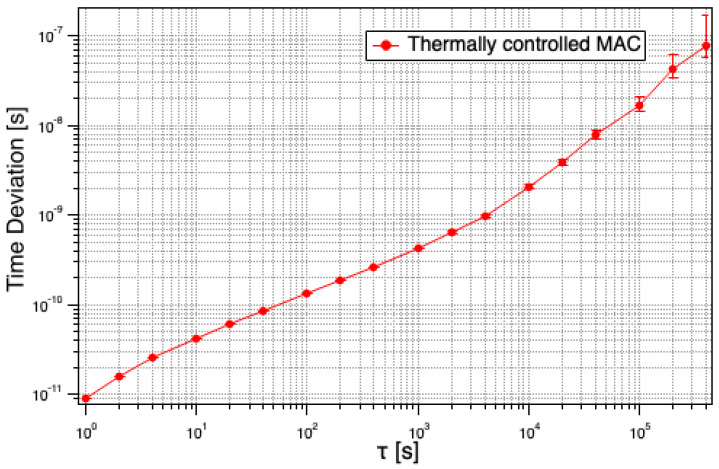
Time Deviation of the MAC achieved with the thermal control, showing better than 2 ns up to 10^4^ s and within 100 ns over four days.

The studies performed in this work demonstrate the capability of a miniature atomic clock as an effective oscillator and its potential as a reference for long holdover ground-based applications and toward space applications that require an excellent long-term stability performance. However, it is important to note that the MAC is yet to be space qualified but has the potential to perform better than the CSAC. Our studies were conducted with the legacy MAC SA.35m and achieved a performance surpassing that of the top-performing commercially available MAC, particularly for the averaging times beyond *τ* > 10^3^ s up to 4 days. Measured Time Deviation of the MAC achieved with thermal control is shown in Figure 5.

The demonstrated MAC stability floor level of <5 × 10^−13^ from 10^4^ s up to four days, with the timing stabilities of <2 ns (up to 10^4^ s) and <100 ns (up to 4 days), signifies the strength of such thermally controlled MAC units as excellent candidates for low SWaP and long holdover ground and LEO applications. We believe the stable behavior seen beyond one day, reaching up to four days, could very well be extended to a week without major changes in the demonstrated design. Additional thermal control studies to gauge further improvement in stability performance of the MAC, utilizing the MAC SA.57m, will inform the development of the thermal isolation of the clock in future models.

Ensuring that the low-SWaP atomic clocks operate in a precisely controlled temperature environment will enable autonomy in small satellite missions with stringent navigation requirements, have the potential to serve as a stable onboard reference in a lunar navigation satellite system and other lunar PNT applications, and augment alternate PNT capabilities of satellites in LEO and MEO. As noted in Section 5, similar methods of thermal control have been incorporated on small satellites and achieve the temperature stability demonstrated in this work.

To realize a reference signal with further enhanced stability, the construction of a clock ensemble consisting of several miniature atomic clocks can be explored. In this, the long-term stability would nearly match that of the best-performing clock in the ensemble but increase the reliability of the overall system. Recently, a testbed of a small clock ensemble with an OCXO and three CSACs was studied and achieved a performance that matched the short-term stability of the OCXO and the long-term stability of the best CSAC in the ensemble [44]. An atomic clock ensemble in space (ACES) will be deployed on the International Space Station that takes advantage of the stabilities of a hydrogen maser and a cesium standard to create a clock signal with exceptional short-term and long-term stability for fundamental physics tests [45]. Similarly, employment of a MAC ensemble with improved thermal control on small satellites will strengthen alternate PNT services by providing a superior short-term and long-term stability over that of a single clock. As this work has shown, amelioration of the thermal sensitivity of the MACs will be critical in achieving a high-quality stability performance, aiding in future PNT based on small satellites for the Earth, Moon, and beyond.

## Figures and Tables

**Table 1:** Coefficient for optimum time error prediction [18].

Noise Type	ADEV Slope	*k*
White and Flicker PM	*τ* ^−1^	$\frac{1}{\sqrt{3}}$
White FM	*τ* ^−1/2^	1
Flicker FM	*τ* ^0^	$\frac{1}{\sqrt{ln2}}$
Random Walk FM	*τ* ^1/2^	1

**Table 3:** Drift and the positioning error induced by drift from ambient and enhanced thermal control.

Characterization Conditions	Drift [per day]	Positioning Error at 24 h (τ = 4 × 10^4^ s) [m]
Ambient	8.68 × 10^−13^	11.3
Enhanced Thermal Control	3.32 × 10^−13^	4.3

**Table 4:** Allan Deviation and related positioning error values from the MAC datasheets and individual characterizations of the ambient performance and thermally controlled performance.

Model	Microchip MAC SA.35m						Microchip MAC SA.57m	
Characterization Conditions	Datasheet		Ambient ^†^		Enhanced Thermal Control ^†^		Datasheet	
Tau [s]	ADEV	Positioning Error [m]	ADEV	Positioning Error [m]	ADEV	Positioning Error [m]	ADEV	Positioning Error [m]
10^1^	1.60 × 10^−11^	0.05	8.80 × 10^−12^	0.03	9.82 × 10^−12^	0.03	5.00 × 10^−12^	0.02
10^2^	8.00 × 10^−12^	0.24	3.12 × 10^−12^	0.09	3.28 × 10^−12^	0.10	1.50 × 10^−12^	0.05
10^3^	N/A *	-	1.16 × 10^−12^	0.42	1.03 × 10^−12^	0.31	5.00 × 10^−13^	0.19
10^4^	N/A *	-	1.98 × 10^−12^	5.94	4.49 × 10^−13^	1.62	1.50 × 10^−12^	4.50
10^5^	N/A *	-	1.62 × 10^−12^	48.6	3.73 × 10^−13^	11.2	1.50 × 10^−12^ **	45.0

^†^ Values taken from individual clock characterization testing. * Values not specified in the manufacturer’s datasheet. ** Values not specified in the manufacturer’s datasheet, assumed ADEV maintained from *τ* = 10^4^ s.

## Data Availability

We The original contributions presented in this study are included in the article. Further inquiries can be directed to the corresponding author.

## Abbreviations

The following abbreviations are used in this manuscript:

SWaP	Size, weight, and power
CSAC	Chip-scale atomic clock
MAC	Miniature atomic clock
PNT	Positioning, navigation, and timing
GNSS	Global Navigation Satellite System
LEO	Low-Earth orbit
ADEV	Allan Deviation
GPS	Global Positioning System
MEO	Medium-Earth orbit
